# Defining the True Sensitivity of Culture for the Diagnosis of Melioidosis Using Bayesian Latent Class Models

**DOI:** 10.1371/journal.pone.0012485

**Published:** 2010-08-30

**Authors:** Direk Limmathurotsakul, Kris Jamsen, Arkhom Arayawichanont, Julie A. Simpson, Lisa J. White, Sue J. Lee, Vanaporn Wuthiekanun, Narisara Chantratita, Allen Cheng, Nicholas P. J. Day, Claudio Verzilli, Sharon J. Peacock

**Affiliations:** 1 Department of Tropical Hygiene, Faculty of Tropical Medicine, Mahidol University, Bangkok, Thailand; 2 Mahidol-Oxford Tropical Medicine Research Unit, Faculty of Tropical Medicine, Mahidol University, Bangkok, Thailand; 3 Centre for Molecular, Environmental, Genetic and Analytic Epidemiology, School of Population Health, University of Melbourne, Carlton, Australia; 4 Department of Medicine, Sappasithiprasong Hospital, Ubon Ratchathani, Thailand; 5 Center for Clinical Vaccinology and Tropical Medicine, Nuffield Department of Clinical Medicine, Churchill Hospital, University of Oxford, Oxford, United Kingdom; 6 Department of Microbiology and Immunology, Faculty of Tropical Medicine, Mahidol University, Bangkok, Thailand; 7 Department of Epidemiology and Preventive Medicine, Monash University, Melbourne, Australia; 8 Menzies School of Health Research, Charles Darwin University, Casuarina, Australia; 9 Department of Epidemiology and Population Health, London School of Hygiene and Tropical Medicine, London, United Kingdom; 10 Department of Medicine, University of Cambridge, Addenbrooke's Hospital, Cambridge, United Kingdom; Direccion General de Epidemiologia, Peru

## Abstract

**Background:**

Culture remains the diagnostic gold standard for many bacterial infections, and the method against which other tests are often evaluated. Specificity of culture is 100% if the pathogenic organism is not found in healthy subjects, but the sensitivity of culture is more difficult to determine and may be low. Here, we apply Bayesian latent class models (LCMs) to data from patients with a single Gram-negative bacterial infection and define the true sensitivity of culture together with the impact of misclassification by culture on the reported accuracy of alternative diagnostic tests.

**Methods/Principal Findings:**

Data from published studies describing the application of five diagnostic tests (culture and four serological tests) to a patient cohort with suspected melioidosis were re-analysed using several Bayesian LCMs. Sensitivities, specificities, and positive and negative predictive values (PPVs and NPVs) were calculated. Of 320 patients with suspected melioidosis, 119 (37%) had culture confirmed melioidosis. Using the final model (Bayesian LCM with conditional dependence between serological tests), the sensitivity of culture was estimated to be 60.2%. Prediction accuracy of the final model was assessed using a classification tool to grade patients according to the likelihood of melioidosis, which indicated that an estimated disease prevalence of 61.6% was credible. Estimates of sensitivities, specificities, PPVs and NPVs of four serological tests were significantly different from previously published values in which culture was used as the gold standard.

**Conclusions/Significance:**

Culture has low sensitivity and low NPV for the diagnosis of melioidosis and is an imperfect gold standard against which to evaluate alternative tests. Models should be used to support the evaluation of diagnostic tests with an imperfect gold standard. It is likely that the poor sensitivity/specificity of culture is not specific for melioidosis, but rather a generic problem for many bacterial and fungal infections.

## Introduction

Culture remains the diagnostic gold standard for many bacterial and fungal infections [1], [2]. Specificity of culture is based on the likelihood that the organism isolated can be found in healthy subjects, and varies between samples taken from normally sterile versus colonised sites as well as the microbial species in question [3]. More problematic is the true sensitivity of culture, which is difficult to determine but may be low. Insights into the extent to which culture is falsely negative can be gained using molecular tests with a higher predicted diagnostic sensitivity, although both culture and molecular tests are prone to reduced sensitivity from factors such as inadequate sampling, the intermittent presence or low number of organisms in specimens such as blood, and prior administration of antimicrobial therapy [1].

Despite its obvious imperfections and often because of the lack of a better alternative, culture may be used as the gold standard against which alternative diagnostic tests for bacterial infectious diseases are evaluated. The impact of using an imperfect gold standard during the evaluation of a second test can be demonstrated using a hypothetical example, in which a population of 1,000 infected subjects and 1,000 non-infected subjects are evaluated using an imperfect gold standard with a true sensitivity of 60% and true specificity of 100%, and a new test with a true sensitivity of 95% and true specificity of 95%. The estimated sensitivity and specificity of the new test under these circumstances would be 95% (570/600) and 69% (970/1,400), respectively. In addition, the estimated prevalence would be 30% (600/2,000) rather than 50%. Hence, the estimates of both test accuracy and prevalence are strongly biased due to disease misclassification by the imperfect gold standard.

Here, we describe the application of Bayesian latent class models (LCM's) to define the true sensitivity of culture for microbial infection, in which we use a single Gram-negative bacterial infection (melioidosis) as a model system. This often life-threatening infection caused by the environmental saprophyte *Burkholderia pseudomallei* occurs across Southeast Asia and northern Australia [4]. The current diagnostic gold standard is culture and isolation of *B. pseudomallei* from any clinical specimen. The specificity of a positive culture is assumed to be 100% since *B. pseudomallei* is not a member of the normal colonizing flora [5], [6], but sensitivity is unlikely to be as high since experienced clinicians commonly make a clinical diagnosis of melioidosis in culture-negative patients. Culture has also been used previously as a gold standard against which alternative diagnostic assays for melioidosis have been evaluated, including several serological tests [7], [8]. These have performed poorly, a finding attributed to high rates of seropositivity in the background population [9]. We have re-analysed existing datasets to define the impact of misclassification by culture on the reported accuracy of these diagnostic tests.

## Methods

### Study patients and diagnostic tests

The data analyzed in this study was generated during two previously published prospective clinical evaluations of diagnostic laboratory tests for melioidosis [7], [8]. The same patient cohort was used in both studies. In brief, patients were recruited between June and October 2004 at the Sappasithiprasong Hospital, Ubon Ratchathani, northeast Thailand [7]. Inclusion criteria were the presence of a fever (>38.5°C) in patients aged 14 years or more who were suspected to have melioidosis in the absence of clinical or laboratory findings suggestive of an alternative diagnosis. Patients underwent sampling for culture (blood from all patients, and urine, pus, respiratory secretions, throat swab, and swabs from surface lesions, as available or clinically appropriate), and were tested using four serological tests (indirect hemagglutination test (IHA), IgM immunochromogenic cassette test (ICT), IgG ICT, and ELISA using affinity-purified antigen), as previously described [7], [8]. The IHA detects antibody to a poorly defined mixture of antigens present in *B. pseudomallei* culture supernatant, the ICT detects specific IgM or IgG antibodies to *B. pseudomallei* [7], and the ELISA detects antibody to affinity-purified *B. pseudomallei* antigen prepared using a monoclonal antibody to *B. pseudomallei* exopolysaccharide [8]. The serum used in the serological tests was taken at the time of hospital admission. Of the 322 patients recruited [7], two cases were enrolled twice and were excluded from this study.

### Ethics Statement

Ethical approval for the cohort study was obtained from the Ministry of Public Health, Royal Government of Thailand, and the Oxford Tropical Research Ethics Committee, UK. Written inform consent was obtained from each subject enrolled into the study [7].

### Statistical analysis

Results of the five diagnostic test results (culture and four serological tests) were analyzed in three ways. First, culture was used as the gold standard reference, and prevalence, sensitivities, specificities, positive and negative predictive values (PPV's and NPV's) for the four serological tests were calculated with exact 95% confidence intervals using the Stata 10.1 statistical software package (Stata Corp., College Station, Tex.). This was comparable to data published previously [7], [8]. Second, a Bayesian latent class model (LCM) with conditional independence between all five tests was used. In brief, the LCM calculated prevalence and sensitivities and specificities of all tests from the observed frequencies of each possible combination of test results and assumed that, in a given patient, the result of any given test was not associated with the result of any other test. Therefore, this model did not assume a single gold standard test but regarded each test as imperfect in diagnosing the true disease status (infected or not infected). The true disease status of the patient population was defined on the basis of overall prevalence. All parameters were estimated with 95% credible intervals using WinBUGS 1.4 (http://www.mrc-bsu.cam.ac.uk/bugs/welcome.shtml) [10]. Third, Bayesian LCM's with conditional dependence between diagnostic tests were used. A class of fixed effect and random effect models described by Dendukuri and Joseph were used to take account of conditional dependence between tests [11], [12]. Fixed effect models were used for pairwise correlation between two tests, and random effect models were used for correlation between more than two tests. On the basis of published knowledge [4], [8], [9], [13], four probable correlations between diagnostic tests were explored (Table S1). For Bayesian LCM's, specificity of culture was fixed at 100%, and we assumed that we knew nothing (non-informative priors) about the unknown parameters (prevalence, sensitivities of all five tests and specificities of all four serological tests). Bayesian p-value, deviance information criteria (DIC) and Akaike's information criterion (AIC) were used to compare the models [14].

### Post-hoc model evaluation

The prediction accuracy of the final model was tested using a clinical tool that was developed to estimate the probability of melioidosis in patients who were culture negative for *B. pseudomallei*. This was based on the following data that was gathered throughout hospital admission to the time of death or discharge: (i) clinical progression, (ii) the results of additional investigations, and (iii) administration and response to antimicrobial therapy, including details of the antimicrobial(s) used and whether this would be effective treatment for melioidosis. Final diagnoses were categorized into 4 groups: (i) definite melioidosis (culture-confirmed), (ii) probable melioidosis, (iii) possible melioidosis, or (iv) melioidosis was unlikely or excluded. Table 1 describes the definitions used for each group.

**Table 1:** Criteria used to determine the possibility of having melioidosis.

Definite melioidosis (Culture confirmed)	One or more clinical samples culture positive for *B. pseudomallei*
Probable melioidosis (Clinical melioidosis)	Presence of multiple liver abscesses and/or single or multiple splenic abscess(es) on abdominal ultrasound with an appearance that is characteristic for melioidosis (swiss cheese appearance or small dispersed abscesses), but culture not performed or negative for *B. pseudomallei * ***OR*** Culture negative for *B. pseudomallei* on first presentation but represented to hospital within one month with culture proven melioidosis
Possible melioidosis (Findings that fall short of ‘probable’ but are not ‘unlikely’)	Clinically suspected melioidosis and improved after treatment with an effective antimicrobial regimen for melioidosis (ceftazidime/carbapenem drug/amoxicillin-clavulanate), ***OR*** Clinically suspected melioidosis and died before improvement observed
Not melioidosis (Melioidosis is unlikely)	Definite alternative diagnosis for manifestations leading to suspected melioidosis, ***OR*** Resolution of clinical features of suspected melioidosis without treatment with antimicrobial drugs with activity against *B. pseudomallei*

## Results

A total of 320 patients with suspected melioidosis were included in the study. The median patient age was 54 years (interquartile range 43–65 years), and 161 patients (50%) were male. 119 out of 320 patients were culture positive for *B. pseudomallei*, giving a prevalence of 37.2% (95% confidence interval 31.9–42.7). The number of patients who were culture-positive for each sample type was as follows: blood, 65 (54.6%); urine, 20 (16.8%); sputum, 37 (31.1%); throat swab, 26 (21.9%); and other specimens, 51 (42.9%); (many patients were positive for more than one sample type). IHA, IgM ICT, IgG ICT and ELISA were positive in 158, 200, 206 and 152 patients, respectively. Sixty-nine patients (21.6%) were positive for culture and all four serological tests.

Using data on clinical progression during the course of hospital admission through to the time of death or hospital discharge, 119/320 patients (37.2%) were defined as having definite (culture confirmed) melioidosis, 34/320 (10.6%) were assigned to the probable group, 83/320 (25.9%) were assigned to the possible group, and 84/320 (26.3%) were assigned to the unlikely/non-melioidosis group. Diabetes was present in 85/119 (71%) patients with definite melioidosis, 21/35 (60%) with probable melioidosis, 49/76 (65%) with possible melioidosis and 49/90 (54%) in the non-melioidosis group (p = 0.08). In the probable group, 33/34 patients were defined as having clinical melioidosis on the basis of hepatosplenic abscess(es) detected by ultrasonogram, and 1 patient re-presented with culture confirmed melioidosis after hospital discharge. In the possible group, 63 patients improved after treatment with effective antimicrobial regimens for melioidosis, and 20 patients died before clinical progression could be observed. In the non-melioidosis group, 44 patients had resolution of clinical features without treatment with antimicrobial drugs that are active against *B. pseudomallei*, and the remaining patients had a range of other diagnoses, as follows: bacteremia with an organism other than *B. pseudomallei* (11), leptospirosis (7), malignancy (7), tuberculosis (5), acute myocardial infarction (3), amoebic liver abscess (1), single liver abscess of unknown cause (1), cholangitis (1), cholecystitis (1), herpes zoster (1), polyarteritis nodosa (1), and congestive heart failure (1).

### Culture as a perfect gold standard

We first assumed that culture was a perfect gold standard (100% sensitivity and 100% specificity), and used this assumption to calculate the sensitivities, specificities, PPV's and NPV's of the four serological tests (Table 2). The ELISA gave the highest combination of sensitivity and specificity (82.4% and 73.1%, respectively). All serological tests lacked specificity, a finding that was most marked for the IgM ICT (48.8%) and IgG ICT (49.3%).

**Table 2:** Prevalence, sensitivities and specificities, positive and negative predictive values (PPV's and NPV's) using culture as gold standard and for two Bayesian latent class models.

Parameters	Culture as gold standard*	Model 0†	Final model†
Prevalence	37.2 (31.9–42.7)	61.0 (54.7–67.2)‡	61.6 (54.4–69.2)‡
Culture			
Sensitivity	100	60.9 (53.3–68.6)	60.2 (51.7–68.5)
Specificity	100	100	100
PPV	100	100	100
NPV	100	62.1 (53.5–70.5)	61.9 (50.0–70.9)
IHA			
Sensitivity	71.4 (63.2–79.7)	73.0 (66.1–79.4)	69.9 (63.6–76.0)
Specificity	63.7 (57.0–70.4)	87.7 (80.0–93.9)	83.9 (74.9–91.4)
PPV	53.8 (45.9–61.7)	90.3 (83.7–95.3)	87.5 (79.4–93.9)
NPV	79.0 (72.7–85.4)	67.4 (58.8–75.5)	63.4 (52.7–72.5)
IgM ICT			
Sensitivity	81.5 (74.4–88.6)	80.4 (74.1–85.8)	77.5 (71.4–83.1)
Specificity	48.8 (41.8–55.7)	65.5 (56.3–74.5)	62.0 (52.0–72.1)
PPV	48.5 (41.5–55.5)	78.5 (71.2–84.9)	76.7 (68.5–84.2)
NPV	81.7 (74.6–88.7)	68.0 (58.4–76.9)	63.2 (51.0–73.4)
IgG ICT			
Sensitivity	87.4 (81.3–93.4)	91.1 (86.3–94.7)	88.0 (82.4–92.4)
Specificity	49.3 (42.3–56.2)	77.5 (67.8–86.4)	74.1 (63.2–85.2)
PPV	50.5 (43.6–57.4)	86.4 (79.3–92.3)	84.5 (76.1–92.2)
NPV	86.8 (80.5–93.1)	84.8 (76.8–91.0)	79.4 (68.4–87.3)
ELISA			
Sensitivity	82.4 (75.4–89.3)	77.1 (69.9–83.8)	75.6 (67.9–82.8)
Specificity	73.1 (67.0–79.3)	99.4 (94.5–100)	97.9 (92.4–99.9)
PPV	64.5 (56.8–72.2)	99.5 (95.4–100)	98.3 (93.7–99.9)
NPV	87.5 (82.4–92.6)	73.5 (64.3–81.6)	71.3 (59.3–81.3)

Model 0 assumed that for a given patient, each diagnostic test was not correlated. The final model (Model 3) assumed that in infected patients, all serological tests were correlated.

* Values shown are mean estimates with 95% confidence interval.

† Values shown are median estimates with 95% credible interval.

‡ Prevalence estimated by the models are for the test population as a whole, since they cannot determine whether a given patient was infected or not infected.

### Conditional independence model

**Figure 1:**
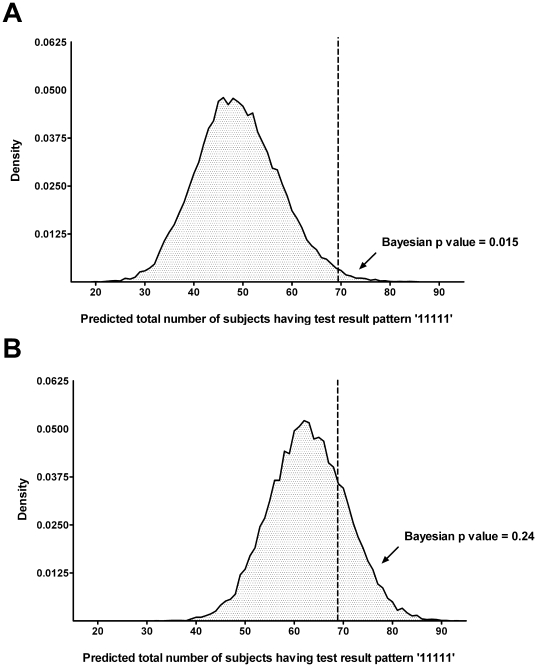
Assessing the fitness of model 0 (conditional independence model) (A) and the final model (conditional dependence model) (B) using probability analysis (posterior predictive distribution). Dataset was replicated for 20,000 times per model to assess the probability that the actual dataset was being observed, if that model was true. Running model 0 a total of 20,000 times (Figure 1A), we found that only 298 replicate datasets had at least 69 patients with all five tests positive and giving the profile ‘11111’ (69 was the number of patients having this profile in the actual dataset) (298/20,000, Bayesian p value 0.015). This indicated that model 0 was not a good fit for the observed data. Running the final model a total of 20,000 times (Figure 1B), we found that 4,752 replicate datasets had at least 69 patients with the profile ‘11111’ (4,752/20,000, Bayesian p value 0.24), indicating that the final model fit the observed data well.

We then assumed that culture might be an imperfect gold standard and applied a conditional independence model to the data for the five tests, which we termed Model 0. Inherent to this model is the assumption that, for a given patient, knowing the result of the first test has no influence on the result of the second test. Similarly, knowing the results of the first and second test has no influence on the result of the remaining tests. The observed frequencies of the 32 possible combinations of results for the 5 tests (from all tests positive giving a profile of 1,1,1,1,1 to all tests negative giving a profile of 0,0,0,0,0) are shown in Table S2. Using the observed frequencies of the 32 possible combinations, we can estimate the sensitivities, specificities, PPV's and NPV's of the 5 diagnostic tests (Table 2). Sensitivity of culture was estimated to be 60.9% (95% Credible Interval 53.3–68.6). Specificities of the other four tests were considerably higher than those estimated using culture as a perfect gold standard. However, the observed frequency of patients having all tests positive was considerably higher than was predicted by the conditional independence model (69 vs. 49 patients; Bayesian p value = 0.015; Table S3, Figure 1a). A Bayesian p value this close to zero indicates that the observed result would be unlikely to be seen in replications of the data if the model was true. This was strongly suggestive of a positive correlation between diagnostic tests where patients who were positive for one test were more likely to be positive for other tests, an observation with biological plausibility. We concluded, therefore, that this conditional independence model was not a good fit for the data.

### Conditional dependence model

We then assumed not only that culture might be imperfect, but also that diagnostic tests could be correlated. Correlations were evaluated between IHA and IgM ICT (Model 1; DIC = 219.4), and between IHA and IgG ICT in infected patients (Model 2; DIC = 198.7). Both models were a significantly better fit than Model 0 (DIC = 233.1), as reflected by the fact that Model 1 and 2 had lower DIC values than Model 0 and that this difference was more than 10 [14]. However, the Bayesian p values of both Model 1 and 2 were still very close to 0 (Table S3), indicating that Model 1 and Model 2 were still not a good fit for the data.

We then assessed the correlation between more than two diagnostic tests by using models with a random effect variable. Correlation among all serological tests in infected patients (Model 3; AIC = 170.5) and correlation among all serological tests in non-infected patients (Model 4; AIC = 226.9) were evaluated. AIC was used rather than DIC, as the DIC of random effect models could not be estimated by WinBUGS [14]. Model 4 did not fit the data better than Model 0 (AIC = 233.7), indicating that correlation of false positivity among serological tests in non-infected patients with high background antibody level was not observed. Model 3 was the best fit for the data (AIC = 170.5, Bayesian p value = 0.24, Figure 1b), and was selected as the final model. WinBUGS code and detail of each model is provided in Text S1 and S2.

### Final model

The prevalence of infected patients in the test population was estimated to be 61.6% (95% confidence interval 54.4%–69.2%), and around 197 (320×61.6%) subjects were classified as having melioidosis. This model indicated that culture had low sensitivity (60.2%, 95% confidence interval 51.7%–68.5%; Table 2). All parameters estimated by the final model were moderately different from those estimated by Model 0 (conditional independence model). This indicated that estimates of parameters could be biased not only by misclassification of the gold standard, but also by failure to take account of conditional dependence between diagnostic tests.

### Post-hoc model validation

The classification of 37.2% of patients into the definite melioidosis group, 10.6% into the probable melioidosis group and 25.9% into the possible melioidosis group means that the true prevalence of melioidosis in the test population could range from 47.8% to 73.7%. This indicates that the estimated prevalence from the final model was credible.

## Discussion

Understanding the sensitivity and specificity of a diagnostic test is central to its appropriate use in clinical practice. Culture is the leading investigation for patients with suspected infection from a wide range of pathogens, but ascertaining the true sensitivity of this test is difficult. Here, we describe an approach to define the sensitivity of culture using melioidosis as a model system, in which Bayesian LCM with conditional dependence gave an estimated sensitivity of 60.2%. Bayesian LCM also gave an estimated prevalence of 61.6% in patients who were investigated for suspected melioidosis, compared with 37.2% based on culture alone. This higher estimated prevalence is credible, since the study was performed at a hospital where melioidosis is the most common cause of community-acquired bacteremia [15], and during the rainy season when most cases of melioidosis occur. These findings have important implications for clinical care. Influenced by the high associated death rate from melioidosis in our setting of 45% [16], we propose that all patients suspected to have this infection should be commenced on empirical intravenous antimicrobials to cover *B. pseudomallei*, and that this be discontinued or changed to another agent only if an alternative diagnosis is made or melioidosis is considered unlikely. The decision to proceed to a course of oral antimicrobial therapy (which is required for 12–20 weeks to eradicate *B. pseudomallei*) should be based on a summary of all available information.

The development of strategies for the evaluation of a diagnostic test when the gold standard used is known to be imperfect has been an active area of biostatistical research applied to many areas including infectious diseases, oncology and veterinary medicine [11], [17]–[20]. Our study has demonstrated that culture represents a poor gold standard against which to compare alternative diagnostic tests for melioidosis, and has shown the utility of statistical models under such circumstances. The shift we observed in calculated diagnostic accuracy of serological tests based on Bayesian LCM compared with previous figures based on the use of culture as gold standard are of sufficient magnitude that some of these tests might now be considered for use in the clinical setting. For example, the ELISA had a PPV and NPV of 64.5% and 87.5%, respectively, when compared with culture and as such had no clinical utility. When re-calculated using Bayesian LCM with conditional dependence, the PPV and NPV were 98.3% and 71.3%, respectively, representing a test that could be used to rule in melioidosis with a high degree of accuracy. No tests had a high NPV in the models used here, and so the clinical problem remains that a diagnosis of melioidosis is difficult to rule out.

Poor sensitivity of culture has several possible explanations. A number of patients received antimicrobials before all clinical specimens could be obtained. The detectable *B. pseudomallei* count in the blood of patients with melioidosis has been reported to be as low as 0.1 CFU/ml [21], and may fall below the level of detection. Sensitivity may also be reduced by the use of non-selective media for samples from colonized sites [22]. Despite these problems, culture of all available clinical specimens is required since microbiological isolation is needed for a definite diagnosis for melioidosis, and a blood or urine culture positive for *B. pseudomallei* is an independent prognostic factor for mortality outcome [23].

Our data supported positive correlations between serological tests in patients with melioidosis. Seropositivity is common in apparently healthy people living in northeast Thailand where contact with *B. pseudomallei* present in the environment is a regular occurrence [24], [25], and we also expected to find that serological tests in non-infected seropositive persons would be correlated, but this was not the case. One possible explanation is that immunological responses occur to a specific subset of bacterial antigens during health, but that infected patients are exposed to a wider range of bacterial antigens. This is consistent with the findings of study that defined immunological responses to *B. pseudomallei* in health and during melioidosis using an immunoarray approach [26].

The development of a standardised tool to assign patients with suspected melioidosis into categories based on variable degrees of diagnostic certainty was an important component of the external model validation. This represents the first description of a systematic grading scheme for melioidosis. We consider it likely that the probable melioidosis and non-melioidosis categories had a high degree of accuracy. All but one patient was assigned to the probable group based on the presence of multiple liver abscesses and/or splenic abscess(es), a feature that has been reported previously to be highly associated with melioidosis in patients presenting with a febrile illness in northeast Thailand [27], [28]. In the non-melioidosis group, a definitive diagnosis was made in most cases, and melioidosis was unlikely to have been the cause of infection in patients without a diagnosis who recovered without antimicrobial therapy with activity against *B. pseudomallei* [29]. The assignment of patients to the possible melioidosis group is likely to be associated with a higher level of uncertainty, since infections caused by other bacterial pathogens may respond well to antimicrobials prescribed for melioidosis.

In conclusion, we consider it likely that the poor sensitivity of culture is not specific for melioidosis, but rather is likely to represent a generic problem of the test. Application of the methodology described here to the evaluation of culture for other infectious diseases would lead to a broader understanding of the utility and limitations of this test. The models described here also represent tools for the future evaluation of diagnostic tests for infectious diseases when the gold standard assay is imperfect.

## Supporting Information

Table S1 Expected correlations between diagnostic tests for melioidosis.
(0.04 MB DOC)

Table S2 Observed and posterior mean predicted frequency of profiles from Bayesian latent class models.
(0.06 MB DOC)

Table S3 Description and model selection criteria.
(0.05 MB DOC)

Text S1 Dataset.
(0.04 MB DOC)

Text S2 WinBUGS models.
(0.06 MB DOC)

## References

[1] Klouche M, Schroder U (2008). Rapid methods for diagnosis of bloodstream infections.. Clin Chem Lab Med.

[2] Alexander BD (2002). Diagnosis of fungal infection: new technologies for the mycology laboratory.. Transpl Infect Dis.

[3] Rogers GB, Carroll MP, Bruce KD (2009). Studying bacterial infections through culture-independent approaches.. J Med Microbiol.

[4] Cheng AC, Currie BJ (2005). Melioidosis: epidemiology, pathophysiology, and management.. Clin Microbiol Rev.

[5] White NJ (2003). Melioidosis.. Lancet.

[6] Wuthiekanun V, Suputtamongkol Y, Simpson AJ, Kanaphun P, White NJ (2001). Value of throat swab in diagnosis of melioidosis.. J Clin Microbiol.

[7] Cheng AC, Peacock SJ, Limmathurotsakul D, Wongsuvan G, Chierakul W (2006). Prospective evaluation of a rapid immunochromogenic cassette test for the diagnosis of melioidosis in northeast Thailand.. Trans R Soc Trop Med Hyg.

[8] Chantratita N, Wuthiekanun V, Thanwisai A, Limmathurotsakul D, Cheng AC (2007). Accuracy of enzyme-linked immunosorbent assay using crude and purified antigens for serodiagnosis of melioidosis.. Clin Vaccine Immunol.

[9] Wuthiekanun V, Chierakul W, Langa S, Chaowagul W, Panpitpat C (2006). Development of antibodies to *Burkholderia pseudomallei* during childhood in melioidosis-endemic northeast Thailand.. Am J Trop Med Hyg.

[10] Lunn D, Thomas A, Best N, Spiegelhalter D (2000). WINBUGS - a Bayesian modelling framework: concepts, structure, and extensibility.. Stat Comput.

[11] Joseph L, Gyorkos TW, Coupal L (1995). Bayesian estimation of disease prevalence and the parameters of diagnostic tests in the absence of a gold standard.. Am J Epidemiol.

[12] Dendukuri N, Joseph L (2001). Bayesian approaches to modeling the conditional dependence between multiple diagnostic tests.. Biometrics.

[13] Wuthiekanun V, Amornchai P, Chierakul W, Cheng AC, White NJ (2004). Evaluation of immunoglobulin M (IgM) and IgG rapid cassette test kits for diagnosis of melioidosis in an area of endemicity.. J Clin Microbiol.

[14] Spiegelhalter D, Best N, Carlin B, Linde A (2009). Bayesian measures of model complexity and fit.. J R Statist Soc B.

[15] Chaowagul W, White NJ, Dance DA, Wattanagoon Y, Naigowit P (1989). Melioidosis: a major cause of community-acquired septicemia in northeastern Thailand.. J Infect Dis.

[16] Limmathurotsakul D, Wongratanacheewin S, Teerawattanasook N, Wongsuvan G, Chaisuksant S (2010). Increasing incidence of human melioidosis in Northeast Thailand.. Am J Trop Med Hyg.

[17] Menten J, Boelaert M, Lesaffre E (2008). Bayesian latent class models with conditionally dependent diagnostic tests: a case study.. Stat Med.

[18] Qu Y, Tan M, Kutner MH (1996). Random effects models in latent class analysis for evaluating accuracy of diagnostic tests.. Biometrics.

[19] Qu Y, Hadgu A (1998). A Model for Evaluating Sensitivity and Specificity for Correlated Diagnostic Tests in Efficacy Studies With an Imperfect Reference Test.. J Am Stat Assoc.

[20] Toft N, Jorgensen E, Hojsgaard S (2005). Diagnosing diagnostic tests: evaluating the assumptions underlying the estimation of sensitivity and specificity in the absence of a gold standard.. Prev Vet Med.

[21] Wuthiekanun V, Limmathurotsakul D, Wongsuvan G, Chierakul W, Teerawattanasook N (2007). Quantitation of *B. Pseudomallei* in clinical samples.. Am J Trop Med Hyg.

[22] Wuthiekanun V, Dance DA, Wattanagoon Y, Supputtamongkol Y, Chaowagul W (1990). The use of selective media for the isolation of *Pseudomonas pseudomallei* in clinical practice.. J Med Microbiol.

[23] Limmathurotsakul D, Wuthiekanun V, Chierakul W, Cheng AC, Maharjan B (2005). Role and significance of quantitative urine cultures in diagnosis of melioidosis.. J Clin Microbiol.

[24] Khupulsup K, Petchclai B (1986). Application of indirect hemagglutination test and indirect fluorescent antibody test for IgM antibody for diagnosis of melioidosis in Thailand.. Am J Trop Med Hyg.

[25] Cheng AC, Wuthiekanun V, Limmathurotsakul D, Chierakul W, Peacock SJ (2008). Intensity of exposure and incidence of melioidosis in Thai children.. Trans R Soc Trop Med Hyg.

[26] Felgner PL, Kayala MA, Vigil A, Burk C, Nakajima-Sasaki R (2009). A *Burkholderia pseudomallei* protein microarray reveals serodiagnostic and cross-reactive antigens.. Proc Natl Acad Sci U S A.

[27] Vatcharapreechasakul T, Suputtamongkol Y, Dance DA, Chaowagul W, White NJ (1992). *Pseudomonas pseudomallei* liver abscesses: a clinical, laboratory, and ultrasonographic study.. Clin Infect Dis.

[28] Sangchan A, Mootsikapun P, Mairiang P (2003). Splenic abscess: clinical features, microbiologic finding, treatment and outcome.. J Med Assoc Thai.

[29] White NJ, Dance DA, Chaowagul W, Wattanagoon Y, Wuthiekanun V (1989). Halving of mortality of severe melioidosis by ceftazidime.. Lancet.

