# Supplementary material for: Defining the True Sensitivity of Culture for the Diagnosis of Melioidosis Using Bayesian Latent Class Models
Source: PLoS One. 2010 Aug 30;5(8):e12485. doi: 10.1371/journal.pone.0012485 (PMC2932979; doi:10.1371/journal.pone.0012485)
Supplement: Table S1 — Expected correlations between diagnostic tests for melioidosis. (0.04 MB DOC) [file pone.0012485.s001.doc]

**Table S1** Expected correlations between diagnostic tests for melioidosis

| **Model** | **Correlation ^*^** | **Scientific Background** |
| --- | --- | --- |
| 1 | IHA and IgM ICT | IHA and IgM ICT are based on antibody detection. Both IHA and IgM ICT are more likely to be positive if IgM is high, and to be negative if IgM is low. |
| 2 | IHA and IgG ICT | IHA and IgG ICT are based on antibody detection. Both IHA and IgG ICT are more likely to be positive if IgG is high, and to be negative if IgG is low. |
| 3 | All serological tests | IHA, IgM ICT, IgG ICT and ELISA are all based on antibody detection. Infected patients with a strong immune response are more likely to have all serological tests positive, whereas infected patients with a poor immune response are more likely to have all serological tests negative. |
| 4 | All serological tests  (non-infected) | IHA, IgM ICT, IgG ICT and ELISA are all based on antibody detection. Non-infected patients with a high background antibody level are more likely to have all serological tests false positive. |

**^*^** All correlations are in infected subjects, unless otherwise specified.
