# Supplementary material for: Defining the True Sensitivity of Culture for the Diagnosis of Melioidosis Using Bayesian Latent Class Models
Source: PLoS One. 2010 Aug 30;5(8):e12485. doi: 10.1371/journal.pone.0012485 (PMC2932979; doi:10.1371/journal.pone.0012485)
Supplement: Table S2 — Observed and posterior mean predicted frequency of profiles from Bayesian latent class models. (0.06 MB DOC) [file pone.0012485.s002.doc]

**Table S2** Observed and posterior mean predicted frequency of profiles from Bayesian latent class models

|  |  | Expected frequency | | | | |
| --- | --- | --- | --- | --- | --- | --- |
| Response profile | Observed  frequency | Model 0 | Model 1 | Model 2 | Model 3 | Model 4 |
| 11111 | 69 | 49 | 53 | 53 | 63 | 49 |
| 11110 | 6 | 15 | 15 | 15 | 7 | 11 |
| 11101 | 0 | 5 | 6 | 1 | 2 | 6 |
| 11100 | 0 | 1 | 2 | 0 | 1 | 1 |
| 11011 | 9 | 12 | 8 | 13 | 6 | 13 |
| 11010 | 0 | 4 | 2 | 4 | 3 | 3 |
| 11001 | 0 | 1 | 1 | 0 | 1 | 2 |
| 11000 | 1 | 0 | 0 | 0 | 1 | 0 |
| 10111 | 14 | 18 | 14 | 14 | 11 | 19 |
| 10110 | 3 | 5 | 4 | 4 | 5 | 4 |
| 10101 | 0 | 2 | 1 | 6 | 1 | 3 |
| 10100 | 5 | 1 | 0 | 2 | 2 | 1 |
| 10011 | 3 | 4 | 9 | 3 | 4 | 5 |
| 10010 | 0 | 1 | 3 | 1 | 5 | 1 |
| 10001 | 3 | 0 | 1 | 2 | 1 | 1 |
| 10000 | 6 | 0 | 0 | 0 | 6 | 0 |
| 01111 | 35 | 31 | 33 | 33 | 42 | 31 |
| 01110 | 15 | 11 | 11 | 11 | 7 | 18 |
| 01101 | 0 | 3 | 4 | 1 | 1 | 3 |
| 01100 | 5 | 5 | 6 | 5 | 6 | 6 |
| 01011 | 5 | 8 | 5 | 8 | 4 | 4 |
| 01010 | 6 | 5 | 4 | 5 | 5 | 5 |
| 01001 | 0 | 1 | 1 | 0 | 1 | 1 |
| 01000 | 7 | 8 | 9 | 8 | 9 | 5 |
| 00111 | 5 | 12 | 9 | 9 | 8 | 9 |
| 00110 | 18 | 12 | 12 | 12 | 13 | 17 |
| 00101 | 0 | 2 | 2 | 5 | 2 | 2 |
| 00100 | 25 | 29 | 29 | 30 | 29 | 19 |
| 00011 | 7 | 3 | 6 | 3 | 3 | 3 |
| 00010 | 11 | 17 | 19 | 18 | 20 | 14 |
| 00001 | 2 | 1 | 2 | 2 | 2 | 2 |
| 00000 | 60 | 55 | 52 | 52 | 50 | 62 |
