# Supplementary material for: Defining the True Sensitivity of Culture for the Diagnosis of Melioidosis Using Bayesian Latent Class Models
Source: PLoS One. 2010 Aug 30;5(8):e12485. doi: 10.1371/journal.pone.0012485 (PMC2932979; doi:10.1371/journal.pone.0012485)
Supplement: Table S3 — Description and model selection criteria. (0.05 MB DOC) [file pone.0012485.s003.doc]

**Table S3** Description and model selection criteria

|  |  |  | Fixed effect model | | | Random effect model | |
| --- | --- | --- | --- | --- | --- | --- | --- |
| Model No | Correlation **^*^** | Number  of parameters **^†^** | pD **^‡^** | DIC **^§^** | Bayesian  p-value **^¶^** | AIC **^**^** | Bayesian  p-value |
| 0 | - | 10 | 9.5 | 233.1 | 0.015 | 233.7 | 0.009 |
| 1 | IHA and IgM ICT | 11 | 10.2 | 219.4 | 0.05 | 220.9 | 0.03 |
| 2 | IHA and IgG ICT | 11 | 9.7 | 198.7 | 0.05 | 201.2 | 0.02 |
| 3 | All serological tests | 11 |  |  |  | 170.5 | 0.24 |
| 4 | All serological tests (non-infected) | 11 |  |  |  | 226.9 | 0.006 |

**^*^** All correlations are in infected subjects, unless otherwise specified.

**^†^ Number of parameters** is the total number of unknown parameters to be estimated. For example, the number 10 for Model 0 represented prevalence (1), sensitivity of culture (1) and sensitivities and specificities (8) of four serological tests. (Specificity of culture is fixed at 100%).

**^‡^ pD** is the effective number of parameters being fitted. Generally, the value of pD is close to the number of unknown parameters in the model.

**^§^ DIC** (deviance information criteria) is a generalization of AIC in a Bayesian setting. DIC was not applicable to the random effect models. The model with the smallest DIC or AIC was estimated to be the model that would best predict a replicate dataset which had the same structure as that actually observed. A difference in DIC of more than 10 indicated definite support to the model with the lower DIC, while a difference of between 5 and 10 was considered substantial, and less than 5 inconclusive [14].

**^¶^ Bayesian p value** is the probability that replicate data from the Bayesian model were more extreme than the observed data. A Bayesian p value close to 0 or 1 indicates that the observed result would be unlikely to be seen in replications of the data if the model was true.

**^**^ AIC** (Akaike information criteria) is the number representing the goodness of fit of an estimated statistical model. Likelihood was calculated from posterior mean predicted frequency of profiles.
