# Supplementary material for: Defining the True Sensitivity of Culture for the Diagnosis of Melioidosis Using Bayesian Latent Class Models
Source: PLoS One. 2010 Aug 30;5(8):e12485. doi: 10.1371/journal.pone.0012485 (PMC2932979; doi:10.1371/journal.pone.0012485)
Supplement: Text S1 — Dataset. (0.04 MB DOC) [file pone.0012485.s004.doc]

**Text S1** Dataset

Two types of the same dataset are provided. The first dataset is grouped data of test result profiles (32 records, Text S1.1) which was used for multinomial models, including fixed effect models. The second data set is test result profile of each individual subject (320 records, Text S1.2) which was used for Bernoulli models, including random effect models.

**Text S1.1** Dataset for multinomial models

These data were used in all multinomial models, including fixed effect models. Variables named a, b, c, d and e denote culture, IHA, IgM ICT, IgG ICT and ELISA, respectively: 1 = positive and 0 = negative test result. Variable ‘freqobs’ (frequent observed) represents the total number of subjects who had the test result profile in that row.

# Data

a[] b[] c[] d[] e[] freqobs[]

1 1 1 1 1 69

1 1 1 1 0 6

1 1 1 0 1 0

1 1 1 0 0 0

1 1 0 1 1 9

1 1 0 1 0 0

1 1 0 0 1 0

1 1 0 0 0 1

1 0 1 1 1 14

1 0 1 1 0 3

1 0 1 0 1 0

1 0 1 0 0 5

1 0 0 1 1 3

1 0 0 1 0 0

1 0 0 0 1 3

1 0 0 0 0 6

0 1 1 1 1 35

0 1 1 1 0 15

0 1 1 0 1 0

0 1 1 0 0 5

0 1 0 1 1 5

0 1 0 1 0 6

0 1 0 0 1 0

0 1 0 0 0 7

0 0 1 1 1 5

0 0 1 1 0 18

0 0 1 0 1 0

0 0 1 0 0 25

0 0 0 1 1 7

0 0 0 1 0 11

0 0 0 0 1 2

0 0 0 0 0 60

END

**Text S1.2** Dataset for Bernoulli models

These data are used in all Bernoulli models, including random effect models. Each column of matrix “y” represents culture, IHA, IgM ICT, IgG ICT and ELISA tests in order, and 1 = positive and 0 = negative test result. Each row represents the result profile for each patient. Variable ‘profile’ represents all 32 possible test result profiles. This is used to calculate the posterior predicted frequency of each profile.

# data

list( list( y=structure(.Data=c(

1, 1, 1, 1, 1,

1, 1, 1, 1, 0,

1, 1, 0, 1, 1,

1, 1, 0, 0, 0,

1, 0, 1, 1, 1,

1, 0, 1, 1, 0,

1, 0, 1, 0, 0,

1, 0, 0, 1, 1,

*(remaining data was omitted as it could be generated from Text S1.1)*

0, 0, 0, 0, 0,

0, 1, 1, 0, 0,

0, 1, 0, 1, 1,

0, 0, 0, 0, 0),.Dim=c(320,5)),

list( profile=structure(.Data=c(

1, 1, 1, 1, 1,

1, 1, 1, 1, 0,

1, 1, 1, 0, 1,

1, 1, 1, 0, 0,

1, 1, 0, 1, 1,

1, 1, 0, 1, 0,

1, 1, 0, 0, 1,

1, 1, 0, 0, 0,

1, 0, 1, 1, 1,

1, 0, 1, 1, 0,

1, 0, 1, 0, 1,

1, 0, 1, 0, 0,

1, 0, 0, 1, 1,

1, 0, 0, 1, 0,

1, 0, 0, 0, 1,

1, 0, 0, 0, 0,

0, 1, 1, 1, 1,

0, 1, 1, 1, 0,

0, 1, 1, 0, 1,

0, 1, 1, 0, 0,

0, 1, 0, 1, 1,

0, 1, 0, 1, 0,

0, 1, 0, 0, 1,

0, 1, 0, 0, 0,

0, 0, 1, 1, 1,

0, 0, 1, 1, 0,

0, 0, 1, 0, 1,

0, 0, 1, 0, 0,

0, 0, 0, 1, 1,

0, 0, 0, 1, 0,

0, 0, 0, 0, 1,

0, 0, 0, 0, 0),.Dim=c(32,5)) )
