# Supplementary material for: Defining the True Sensitivity of Culture for the Diagnosis of Melioidosis Using Bayesian Latent Class Models
Source: PLoS One. 2010 Aug 30;5(8):e12485. doi: 10.1371/journal.pone.0012485 (PMC2932979; doi:10.1371/journal.pone.0012485)
Supplement: Text S2 — WinBUGS models. (0.06 MB DOC) [file pone.0012485.s005.doc]

**Text S2** WinBUGS models

**Text S2.1** WinBUGS code for Model 0 (Multinomial model)

# Multinomial model – conditional independence model (result of each diagnostic test on a given patient was independent conditional on true disease status of the patient)

# Model 0

model{

# Likelihood

freqobs[1:32]~dmulti(p[1:32],320)

for (i in 1:32){

p[i]<-prev*(positive[i])+(1-prev)*(negative[i])

positive[i]<-(s[1]*a[i]+(1-s[1])*(1-a[i])) * (s[2]*b[i]+(1-s[2])*(1-b[i]))

* (s[3]*c[i]+(1-s[3])*(1-c[i])) * (s[4]*d[i]+(1-s[4])*(1-d[i]))

* (s[5]*e[i]+(1-s[5])*(1-e[i]))

negative[i]<-((1-x[1])*a[i]+x[1]*(1-a[i])) * ((1-x[2])*b[i]+x[2]*(1-b[i]))

* ((1-x[3])*c[i]+x[3]*(1-c[i])) * ((1-x[4])*d[i]+x[4]*(1-d[i]))

* ((1-x[5])*e[i]+x[5]*(1-e[i]))

}

# Prior

prev~dbeta(1,1)

s[1]~dbeta(1,1)

x[1]<-1.0

for (j in 2:5){

s[j]~dbeta(1,1)

x[j]~dbeta(1,1)

}

# PPV and NPV

for (j in 1:5){

ppv[j]<- s[j]*prev / (s[j]*prev + (1-x[j])*(1-prev))

npv[j]<- x[j]*(1-prev) / (x[j]*(1-prev) + (1-s[j])*(prev))

}

# Prediction

freqpred[1:32]~dmulti(p[1:32],320)

# Bayesian p value

pvalue<-step(freqpred[1]-69)

}

# Data (Text S1.1)

**Text S2.2** WinBUGS code for Model 0 (Bernoullie model)

# Bernoullie model – conditional independence model (result of each diagnostic test on a given patient was independent conditional on true disease status of the patient)

# Model 0

model{

# Likelihood

for (i in 1:320){

status[i]~dbern(prev)

for(j in 1:5){

y[i,j]~dbern(p[i,j])

ypred[i,j]~dbern(p[i,j])

logit(p[i,j])<-status[i]*alpha[j]+(1-status[i])*beta[j]

}

}

# Prior

prev~dbeta(1,1)

alpha[1]~dnorm(0.0,0.01)I(-1,)

logit(s[1])<-alpha[1]

beta[1]<--1000

x[1]<-1

for (j in 2:5){

alpha[j]~dnorm(0.0,0.01)I(-1,)

beta[j]~dnorm(0.0,0.01)I(,1)

logit(s[j])<-alpha[j]

logit(x[j])<--beta[j]

}

# Prediction

for (i in 1:320) {

for(k in 1:32){

for (j in 1:5){

arraymatched[i,k,j]<-equals(ypred[i,j],profile[k,j])

}

nmatched[i,k]<-sum(arraymatched[i,k,])

matchedprofile[i,k]<-equals(nmatched[i,k],5)

}

}

for(k in 1:32){

freqpred[k]<-sum(matchedprofile[,k])

}

# PPV and NPV

for (j in 1:5){

ppv[j]<- s[j]*prev / (s[j]*prev + (1-x[j])*(1-prev))

npv[j]<- x[j]*(1-prev) / (x[j]*(1-prev) + (1-s[j])*(prev))

}

# Bayesian p value

pvalue<-step(freqpred[1]-69)

}

# Data (Text S1.2)

**Text S2.3** WinBUGS code for Model 1 (Multinomial model with fixed effect)

# Multinomial model – conditional dependence between IHA and IgM ICT (results of IHA and IgM ICT of infected patients were correlated)

# Model 1 (Fixed effect)

model{

# Likelihood

freqobs[1:32]~dmulti(p[1:32],320)

for (i in 1:32){

p[i]<-prev*(positive[i])+(1-prev)*(negative[i])

positive[i]<- (s[1]*a[i]+(1-s[1])*(1-a[i]))

* ((s[2]*b[i]+(1-s[2])*(1-b[i])) * (s[3]*c[i]+(1-s[3])*(1-c[i]))

+ equals(b[i],c[i])*cov23 + (1-equals(b[i],c[i]))*(-cov23))

* (s[4]*d[i]+(1-s[4])*(1-d[i])) * (s[5]*e[i]+(1-s[5])*(1-e[i]))

negative[i]<- ((1-x[1])*a[i]+x[1]*(1-a[i])) * ((1-x[2])*b[i]+x[2]*(1-b[i]))

* ((1-x[3])*c[i]+x[3]*(1-c[i])) * ((1-x[4])*d[i]+x[4]*(1-d[i]))

* ((1-x[5])*e[i]+x[5]*(1-e[i]))

}

# Prior

prev~dbeta(1,1)

s[1]~dbeta(1,1)

x[1]<-1.0

for (j in 2:5){

s[j]~dbeta(1,1)

x[j]~dbeta(1,1)

}

# Prior for the correlation

ub23<-min(s[2],s[3])-s[2]*s[3]

cov23~dunif(0,ub23)

rho23<-cov23/sqrt((s[2])*(1-s[2])*(s[3])*(1-s[3]))

# PPV and NPV (similar to Text S2.1)

# Prediction (similar to Text S2.1)

# Bayesian p value (similar to Text S2.1)

}

# Data (Text S1.1)

**Text S2.4** WinBUGS code for Model 1 (Bernoulli model with random effect)

# Bernoulli model – conditional dependence between IHA and IgM ICT (results of IHA and IgM ICT of infected patients were correlated)

# Model 1 (Random effect)

# Random effect variable represented different IgM level in each infected subject

model{

# Likelihood

for (i in 1:320){

status[i]~dbern(prev)

for(j in 1:5){

y[i,j]~dbern(p[i,j])

ypred[i,j]~dbern(p[i,j])

}

}

for (i in 1:320){

logit(p[i,1])<-status[i]*alpha[1]+(1-status[i])*beta[1]

for(j in 2:3){

logit(p[i,j])<-status[i]*alpha[j]+(1-status[i])*beta[j]+status[i]*IgM*re[i]

logit(pstatus[i,j])<-status[i]*alpha[j]+status[i]*IgM*re[i]+(1-status[i])*(-1000)

}

for(j in 4:5){

logit(p[i,j])<-status[i]*alpha[j]+(1-status[i])*beta[j]

}

re[i]~dnorm(0,1)

}

# Prior

prev~dbeta(1,1)

IgM~dnorm(0,0.01)I(0,)

s[1]~dbeta(1,1)

alpha[1]<-logit(s[1])

beta[1]<--1000

x[1]<-1

for (j in 2:5){

s[j]~dbeta(1,1)

x[j]~dbeta(1,1)

alpha[j]<-logit(s[j])

beta[j]<--logit(x[j])

}

# PPV and NPV

for (j in 2:3){

se[j]<-sum(pstatus[,j]) / sum(status[])

}

se[1]<-s[1]

se[4]<-s[4]

se[5]<-s[5]

for (j in 1:5){

ppv[j]<- se[j]*prev / (se[j]*prev + (1-x[j])*(1-prev))

npv[j]<- x[j]*(1-prev) / (x[j]*(1-prev) + (1-se[j])*(prev))

}

# Prediction (similar to Text S2.2)

# Bayesian p value (similar to Text S2.2)

}

# Data (Text S1.2)

**Text S2.5** WinBUGS code for Model 2 (Multinomial model with fixed effect)

# Multinomial model – conditional dependence between IHA and IgG ICT (results of IHA and IgG ICT of infected patients were correlated)

# Model 2 (Fixed effect)

model{

# Likelihood

freqobs[1:32]~dmulti(p[1:32],320)

for (i in 1:32){

p[i]<-prev*(positive[i])+(1-prev)*(negative[i])

positive[i]<- (s[1]*a[i]+(1-s[1])*(1-a[i])) * (s[3]*c[i]+(1-s[3])*(1-c[i]))

* ((s[2]*b[i]+(1-s[2])*(1-b[i])) * (s[4]*d[i]+(1-s[4])*(1-d[i]))

+ equals(b[i],d[i])*cov24+(1-equals(b[i],d[i]))*(-cov24))

* (s[5]*e[i]+(1-s[5])*(1-e[i]))

negative[i]<- ((1-x[1])*a[i]+x[1]*(1-a[i])) * ((1-x[2])*b[i]+x[2]*(1-b[i]))

* ((1-x[3])*c[i]+x[3]*(1-c[i])) * ((1-x[4])*d[i]+x[4]*(1-d[i]))

* ((1-x[5])*e[i]+x[5]*(1-e[i]))

}

# Prior

prev~dbeta(1,1)

s[1]~dbeta(1,1)

x[1]<-1.0

for (j in 2:5){

s[j]~dbeta(1,1)

x[j]~dbeta(1,1)

}

# Prior for the correlation

ub24<-min(s[2],s[4])-s[2]*s[4]

cov24~dunif(0,ub24)

rho24<-cov24/sqrt(s[2]*s[4]*(1-s[2])*(1-s[4]))

# PPV and NPV (similar to Text S2.1)

# Prediction (similar to Text S2.1)

# Bayesian p value (similar to Text S2.1)

}

# Data (Text S1.1)

**Text S2.6** WinBUGS code for Model 2 (Bernoulli model with random effect)

# Bernoulli model – conditional dependence between IHA and IgG ICT (results of IHA and IgG ICT of infected patients were correlated)

# Model 2 (Random effect)

# Random effect variable represented different IgG level in each infected subject

model{

# Likelihood

for (i in 1:320){

status[i]~dbern(prev)

for(j in 1:5){

y[i,j]~dbern(p[i,j])

# For Prediction

ypred[i,j]~dbern(p[i,j])

}

}

for (i in 1:320){

logit(p[i,1])<-status[i]*alpha[1]+(1-status[i])*beta[1]

logit(p[i,2])<-status[i]*alpha[2]+(1-status[i])*beta[2] +status[i]*IgG*re[i]

logit(p[i,3])<-status[i]*alpha[3]+(1-status[i])*beta[3]

logit(p[i,4])<-status[i]*alpha[4]+(1-status[i])*beta[4] +status[i]*IgG*re[i]

logit(p[i,5])<-status[i]*alpha[5]+(1-status[i])*beta[5]

re[i]~dnorm(0,1)

logit(pstatus[i,2])<-status[i]*alpha[2]+status[i]*IgG*re[i]+(1-status[i])*(-1000)

logit(pstatus[i,4])<-status[i]*alpha[4]+status[i]*IgG*re[i]+(1-status[i])*(-1000)

}

# Prior

prev~dbeta(1,1)

IgG~dnorm(0,0.01)I(0,)

s[1]~dbeta(1,1)

alpha[1]<-logit(s[1])

beta[1]<--1000

x[1]<-1

for (j in 2:5){

s[j]~dbeta(1,1)

x[j]~dbeta(1,1)

alpha[j]<-logit(s[j])

beta[j]<--logit(x[j])

}

# PPV and NPV

se[1]<-s[1]

se[2]<-sum(pstatus[,2]) / sum(status[])

se[3]<-s[3]

se[4]<-sum(pstatus[,4]) / sum(status[])

se[5]<-s[5]

for (j in 1:5){

ppv[j]<- se[j]*prev / (se[j]*prev + (1-x[j])*(1-prev))

npv[j]<- x[j]*(1-prev) / (x[j]*(1-prev) + (1-se[j])*(prev))

}

# Prediction (similar to Text S2.2)

# Bayesian p value (similar to Text S2.2)

}

# Data (Text S1.2)

**Text S2.7** WinBUGS code for Model 3 (Bernoulli model with random effect)

# Bernoulli model – conditional dependence between all serological tests (results of IHA, IgM ICT, IgG ICT and ELISA of infected patients were correlated)

# Model 3 (Random effect)

# Random effect variable represented different antibody level in each infected subject

model{

# Likelihood

for (i in 1:320){

status[i]~dbern(prev)

for(j in 1:5){

y[i,j]~dbern(p[i,j])

# For Prediction

ypred[i,j]~dbern(p[i,j])

}

}

for (i in 1:320){

logit(p[i,1])<-status[i]*alpha[1]+(1-status[i])*beta[1]

for (j in 2:5){

logit(p[i,j])<-status[i]*alpha[j]+(1-status[i])*beta[j]+status[i]*Ab*re[i]

logit(pstatus[i,j])<-status[i]*alpha[j]+status[i]*Ab*re[i]+(1-status[i])*(-1000)

}

re[i]~dnorm(0,1)

}

# Prior

prev~dbeta(1,1)

Ab~dnorm(0,0.01)I(0,)

s[1]~dbeta(1,1)

alpha[1]<-logit(s[1])

beta[1]<--1000

x[1]<-1

for (j in 2:5){

s[j]~dbeta(1,1)

x[j]~dbeta(1,1)

alpha[j]<-logit(s[j])

beta[j]<--logit(x[j])

}

# PPV and NPV

se[1]<-s[1]

for (j in 2:5){

se[j]<-sum(pstatus[,j]) / sum(status[])

}

for (j in 1:5){

ppv[j]<- se[j]*prev / (se[j]*prev + (1-x[j])*(1-prev))

npv[j]<- x[j]*(1-prev) / (x[j]*(1-prev) + (1-se[j])*(prev))

}

# Prediction (similar to Text S2.2)

# Bayesian p value (similar to Text S2.2)

}

# Data (Text S1.2)

**Text S2.8** WinBUGS code for Model 4 (Bernoulli model with random effect)

# Bernoulli model – conditional dependence between all serological tests (results of IHA, IgM ICT, IgG ICT and ELISA of non-infected patients were correlated)

# Model 4 (Random effect)

# Random effect variable represented different antibody level in each non-infected subject

model{

# Likelihood

for (i in 1:320){

status[i]~dbern(prev)

for(j in 1:5){

y[i,j]~dbern(p[i,j])

# For Prediction

ypred[i,j]~dbern(p[i,j])

}

}

for (i in 1:320){

logit(p[i,1])<-status[i]*alpha[1]+(1-status[i])*beta[1]

for (j in 2:5){

logit(p[i,j])<-status[i]*alpha[j]+(1-status[i])*beta[j]+(1-status[i])*Ab*re[i]

logit(pstatus[i,j])<-(1-status[i])*beta[j]+(1-status[i])*Ab*re[i]+status[i]*(-1000)

}

re[i]~dnorm(0,1)

}

# Prior

prev~dbeta(1,1)

Ab~dnorm(0,0.01)I(0,)

s[1]~dbeta(1,1)

alpha[1]<-logit(s[1])

beta[1]<--1000

x[1]<-1

for (j in 2:5){

s[j]~dbeta(1,1)

x[j]~dbeta(1,1)

alpha[j]<-logit(s[j])

beta[j]<--logit(x[j])

}

# PPV and NPV

xp[1]<-x[1]

for (j in 2:5){

xp[j]<-1-sum(pstatus[,j]) / (320-sum(status[]))

}

for (j in 1:5){

ppv[j]<- s[j]*prev / (s[j]*prev + (1-xp[j])*(1-prev))

npv[j]<- xp[j]*(1-prev) / (xp[j]*(1-prev) + (1-s[j])*(prev))

}

# Prediction (similar to Text S2.2)

# Bayesian p value (similar to Text S2.2)

}

# Data (Text S1.2)
